# Association between Female Reproductive Health and Mancozeb: Systematic Review of Experimental Models

**DOI:** 10.3390/ijerph17072580

**Published:** 2020-04-09

**Authors:** Serena Bianchi, Stefania Annarita Nottola, Diana Torge, Maria Grazia Palmerini, Stefano Necozione, Guido Macchiarelli

**Affiliations:** 1 Department of Life, Health and Environmental Sciences, University of L’Aquila, 67100 L’Aquila, Italy; serena.bianchi@univaq.it (S.B.); diana.torge@graduate.univaq.it (D.T.); mariagrazia.palmerini@univaq.it (M.G.P.); stefano.necozione@univaq.it (S.N.); guido.macchiarelli@univaq.it (G.M.); 2 Department of Anatomy, Histology, Forensic Medicine and Orthopaedics, La Sapienza University of Rome, 00161 Rome, Italy

**Keywords:** mancozeb, female reproduction, fertility, systematic review

## Abstract

Mancozeb is a widely used fungicide approved for use in agriculture in many countries with long persistence in the environment and consequent bioaccumulation in tissues and biological fluids. Despite the large amount of studies published in recent years, the relationship between mancozeb exposure and female reproductive health is not fully elucidated. In order to summarize current evidence on mancozeb exposure and female reproductive disease, we performed a systematic review of literature. Preferred Reporting Items for Systematic Reviews and Meta-Analyses (PRISMA) guidelines were used to make this review. An adapted version of the National Toxicology Program’s Office of Health and Assessment and Translation (OHAT) framework was used to evaluate the risk of bias. Electronic search on two databases (PubMed and Scopus) was used to find experimental studies (in vitro and in vivo) on mancozeb exposure. The database search identified 250 scientific articles, 20 of which met our inclusion criteria. Selected data were then reviewed and summarized in tables. Overall, mancozeb represents a hazard for female reproductive health, with different mechanisms of action. Undoubtedly more experimental and epidemiological studies are required to definitively validate mancozeb as reproductive toxicant.

## 1. Introduction

Nowadays female subfertility is a widespread health concern [1,2,3,4,5,6]. In fact, up to one in six couples in Western countries is affected by infertility [7]. The most important risk factors in the development of female subfertility include: the age of the woman [6], ovulatory disorders, chromosomal abnormalities, and defective male fertility [8,9,10]. A widespread variety of environmental pollutants, such as several classes of pesticides, heavy metals, and air particulate matter plays a key role in the pathogenesis of female infertility [8,11,12].

Indeed, female fertility and reproductive health are sensitive to toxic exposure, specifically to endocrine disruptor pollutants [13], and have long-term adverse effects. However, several studies have analyzed the connection between environmental air pollution and female reproductive competence, suggesting an adverse linkage between fertility and toxicants [14].

Among environmental pollutants an important role is played by persistent organic pollutants (POPs), whose application can cause adverse health effects in animal models and humans [15,16,17]. Different types of POPs are widely used in agriculture, and present a very strong persistence, especially on leaves, with bioaccumulation in the food web [15,16]. Pesticides act as endocrine disruptor chemicals, possibly leading to low fecundability, miscarriage, preeclampsia, polycystic ovary syndrome (PCOS), endometriosis, and alterations in the menstrual cycle [15,16,18]. Thus, pesticides may negatively affect female reproductive competence both in adulthood and during embryonic development [19,20].

One of the most important endocrine disruptors, with a wide range of agricultural and industrial applications, is mancozeb. Mancozeb (ethylene bis-dithiocarbamate fungicide (EBCD)) was first registered in the United States in 1948 [21] and introduced in the global fungicide market in 1962 [22]. Mancozeb fungicidal efficacy has been applied in different agricultural and industrial contexts, for example in major agricultural crops (tomato, potato, grapevine), and its use will likely increase by the 2020s due to its low price, global demand for fruits and vegetables, and non-selective fungicidal efficacy [21]. In this context, the main source of exposure to mancozeb is the assumption of contaminated products (e.g., tomatoes, potatoes, citrus fruits) or drinking water [21], while occupational exposure includes inhalation, accidental ingestion, and dermal contact of the fungicide, among industrial and agricultural workers [21].

Mancozeb exposure induces a wide range of environmental hazard, such as Parkinson-like symptoms, thyroid hormone dysfunctions, and defects in fetal development [21,23]. As confirmed by recent studies, mancozeb exposure is a risk factor for spontaneous abortion, maternal mortality, and fetal malformations in rat and rabbit experimental models [21]. Moreover, it is strongly linked to teratogenic, mutagenic, and carcinogenic risks [24] because of its ability to induce genotoxic and malignant alterations in human ovarian cells [21,25,26].

For these reasons, we performed a review of scientific literature to summarize current evidence of the influence of mancozeb on female reproductive health. This systematic approach is useful to understand mancozeb’s mechanism of action and role for reproductive and overall female health.

## 2. Materials and Methods

### 2.1. Search Strategy

This systematic review screened PubMed (www.ncbi.nlm.nih.gov) and Scopus (https://www.scopus.com/standard/marketing.uri) databases to select high-profile studies. Studies dealing with in vitro models were screened from January 2002 to December 2019. Studies dealing with mammalian models were screened from January 1973 to December 2019.

### 2.2. Search Terms

A wide range of keywords were used: “Mancozeb” or “ethylene thiourea (ETU)” or “dithiocarbamates” and (1) “reproductive effect” or “infertility” or “reproductive toxicity”; (2) “birth outcomes” or “pregnancy” or “chronic exposure”. This electronic search combined terms and descriptions linked to mancozeb exposure and female reproductive health. We also screened the related references of all relevant articles and overviews.

### 2.3. Inclusion and Exclusion Criteria

We included in vitro studies, with increasing concentrations of mancozeb added in cell culture and in vivo studies with oral or injected mancozeb administration, to assess the effect of mancozeb on female reproductive competence. The exclusion criteria of this systematic review were: (1) no peer-review (e.g., review articles and editorials excluded); (2) lack of reproductive and health outcomes; (3) lack of data on mancozeb exposure; (4) human study population; (5) male study population; and (6) non-English articles. The included articles evaluated the association between mancozeb (or agrochemicals mixtures) exposure and reproductive female health.

### 2.4. Study Selection

Two independent authors (S.B. and S.A.N.) dealt with the primary literature research. The same researchers conducted a second re-evaluation of the selected titles in which the studies not adapting to the established eligibility and inclusion criteria were deleted. Therefore, the remaining reports were deeply screened considering the full-text articles for compatibility. In case of any disagreement between the authors after independent evaluation, consensus was reached by re-evaluation and discussion.

In the event of discrepancies in the data, when possible, reference paper authors were contacted by email for further explanation. The remaining studies were finally reviewed for qualitative synthesis.

We adhered to the Preferred Reporting Items for Systematic Reviews and Meta-Analyses (PRISMA) guidelines [27].

### 2.5. Data and Quality of Data Evaluation Strategy

We adhered to the National Toxicology Program’s Office for Health Assessment and Translation (OHAT) systematic review framework [28] to evaluate mancozeb reproductive toxicity. The internal validity of the included studies was assessed by the OHAT Risk of Bias Rating Tool [29,30]. Confidence ratings were evaluated for each included study considering the study design (Table 1).

## 3. Results

**Figure 1:**
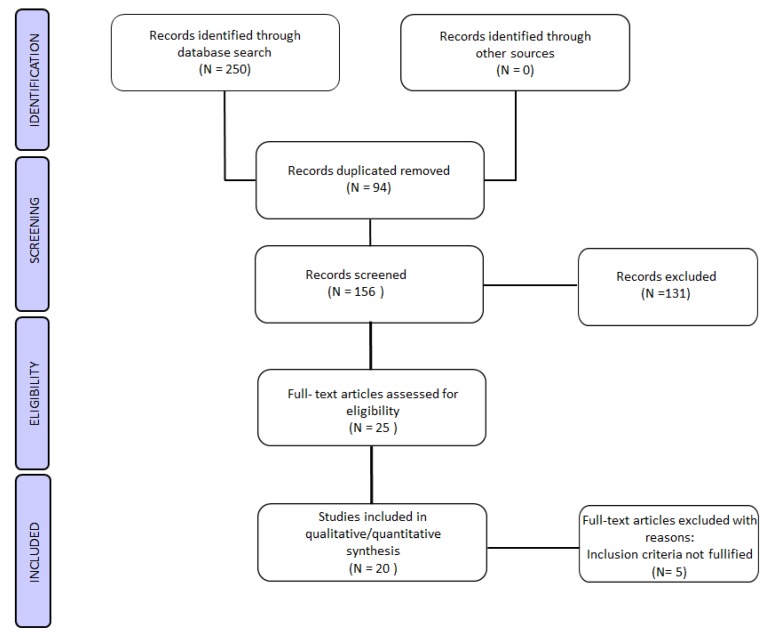
Flow chart of the experimental study selection process.

After literature screening, a total of twenty studies were found eligible for the qualitative synthesis. Among them, seven in vitro studies were found eligible and thirteen studies using mammalian models were found meeting our inclusion criteria (Figure 1).

### 3.1. In Vitro Experimental Studies

Seven in vitro studies, published between 2002 and 2018, met our inclusion criteria and examined mancozeb toxicological and reproductive effects on female subjects. Six studies used mammalian cells in the study design, and all the included in vitro studies demonstrated mancozeb’s power to disrupt female reproduction from a cellular to a molecular and toxicological point of view (Table 2).

Fejes et al. suggested the absence of toxic effects on chicken embryos after 19 days of mancozeb administration (dithane M-45, 80% mancozeb). Instead, an increased mortality on chicken embryos was registered from combined exposure to mancozeb and copper-sulfate (0.1% concentration), when compared to individual doses [31]. Greenlee et al. found a decrease in mouse blastocyst development after low-dose exposure to mancozeb (0.003 µg/mL), with increased apoptosis during blastocyst formation [32].

Mancozeb (1.0, 2.5, and 5.0 µg/mL) also affects cytoplasmic and nuclear maturation during in vitro maturation in buffalo oocytes, inducing a dose-dependent oocyte degeneration [33]. Abdoon et al. also reported the presence of fragmented zona pellucida and cytoplasm in buffalo oocytes [33]. Instead, during in vitro fertilization, a reduction in embryo development (to morula and blastocyst stage) occurred, with cytoplasm degeneration, in all the exposed embryos [33].

Paro et al. showed how increasing concentrations of mancozeb (0.001–1 µg/mL) induce alterations in morphology and migration patterns in mouse granulosa cells, with a reduction in p53 expression levels [25]. Human granulosa cells, derived from women who underwent assisted reproductive therapy, when exposed to increasing mancozeb concentrations, showed alteration in p53 levels and morphology changes. Reduced expression of p53, due to mancozeb administration (0.01 µg/mL), was also confirmed by Iorio et al. in mouse granulosa cells [34] during 36 h of incubation. They also found a depolarization in mitochondrial membrane and a reduction in ATP levels. At the same time, a decrease in glutathione (GSH) levels occurred with an increase in reactive oxygen species (ROS) production [34]. Dose-dependent mancozeb toxicity on reproductive female health was also confirmed by Palmerini et al. who showed mancozeb’s potential to induce ultrastructural and cellular alterations in mouse granulosa cells exposed to increasing concentrations of this fungicide (0.001, 0.01, and 1 µg/mL) [22]. They also highlighted the association between mancozeb administration intercellular contact alteration and chromatin marginalization, phenomena strongly linked with apoptosis and cellular degeneration [22].

Finally, Atmaca et al. demonstrated how mancozeb exposure (1 µM) determines a significant decline in steroids synthesis (day 3 and 5) in bovine luteal cells, when compared to controls [35].

### 3.2. In Vivo Experimental Studies

Thirteen studies with various experimental models (rats, mice, and rabbits), published between 1973 and 2019, adhered to our inclusion criteria. In these experimental studies, mancozeb was administered orally, via gavage, in drinking water and mixed into the diet. Two studies assessed mancozeb exposure, when mixed with other agrochemicals compounds (Table 3).

To this aim, Khera et al. showed that oral ETU administration (0.5, 10, 20, 40, or 80 mg/kg/day), on adult female Wistar rats and New Zealand White rabbits had no significant effects before and during pregnancy [36]. In fact, no maternal toxicity or fetal death in offspring was reported. In addition, Castro et al. confirmed the absence of menstrual cycle alteration in Wister rats exposed to mancozeb concentrations. At the same time, mancozeb do not influence the number of live pup births [37].

Studies conducted on female virgin Wistar rats demonstrated that oral mancozeb administration (500, 600, 700, and 800 mg/kg/day) induces a reduction in ovary enlargement (highest doses: 700–800 mg/kg/day) and does not affect the estrous cycle (500 mg/kg/day). In this context, Mahadevaswami et al. found a huge decline in follicles counts and an increase in defective ovarian follicles (800 mg/kg/day) [38]. Baligar and Kaliwal, instead, found that mancozeb oral exposure (500, 600, 700, and 800 mg/kg/day), in the same experimental model, induces a progressive decline in the number of healthy follicles and significant changes in the estrous cycle (proestrus, estrous, and metestrus phases) [39]. These data are confirmed by the same author in a 2004 study, where mancozeb exposure (700 mg/kg/day) in female virgin albino rats was associated to the progressive decline of healthy follicles, more atretic follicles, and alteration in the estrous cycle [40].

Higher exposure of mancozeb (500 mg/kg) in the prenatal period is linked to oocytes degeneration and a decrease in fertilization competence [41]. Hass et al., instead, suggested that mancozeb exposure (highest concentration: 25 mg/kg/day) induces a longer gestation period in female Wistar rats [42]. Other studies demonstrated that mancozeb administration (via gavage; mixture composition, 6.25, 12.5, 18.75, 25.0, and 31.25 mg/kg/day) does not determine changes in uterus and ovary weight [43], even if there are more perinatal deaths and impaired parturition [44].

Oral mancozeb exposure (800 mg/kg/day) also reduces litter size and weight with a relevant decrease in ovary weight [45]. In this context, there was also an increase in atretic follicles and a reduction in healthy oocytes. Liu et al. also reported ultrastructural changes in Germinal Vesicle (GV) oocytes due to mancozeb administration [45]. Noteworthy, is the decrease in the number of pronuclei and two-cells of parthenogenetic activated oocytes and the alteration in actin expression levels [45]. There is an increase in apoptotic pathways and ROS production, with an abnormal mitochondrial distribution. Finally, mancozeb exposure is strongly linked to epigenetic modifications, which can compromise female fecundability [45]. Other studies suggest the potential of ethylene thiourea to influence important biomarkers of ovarian aging, in CD-1 mice exposed to 0.1, 1, and 10 mg/kg/day, from conception to postnatal day 21 [46].

Among our most recent data, Mahdi et al. showed that gavage mancozeb administration (500 mg/kg), in first generation mouse female pups, induces depletion of germ cells in female gonads with the presence of more atretic follicles [47]. Esmaiel et al. reported a decrease in the number of collected oocytes and a defective maturation, fertilization, and implantation process after mancozeb treatment (gavage; 500 vmg/kg) [48]. Esmaiel et al. also found defective embryo development in their experimental model as well as a compromised fecundity rate.

## 4. Discussion

The studies included in this systematic review, published between 1973 and 2019, confirm that lower and higher mancozeb concentrations, individually or administered in combination with other agrochemical compounds, compromise female reproductive health.

High and moderate levels of confidence, as regards in vitro studies, prove the ability of mancozeb to affect, directly or indirectly, female reproductive competence, by exerting its toxicity in the cellular environment from an ultrastructural to a molecular point of view.

To this aim, Abdoon et al. suggested that mancozeb induces a dose-dependent degeneration process in buffalo oocytes during in vitro maturation [33]. Mancozeb exposure indeed affects cytoplasmic and nuclear maturation: this fungicide reduces the oocyte nuclear maturation, with a direct effect on female reproductive cells [33]. At the same time, mancozeb exposure influences embryo development, especially in the morula and blastocyst stages. In this context, embryos exposed to different concentrations of mancozeb show fragmented and degenerated cytoplasm [33]. In addition, Greenlee et al. reported a significant decrease in mouse blastocyst development [32]; conversely, Fejes et al. found that mancozeb has no toxic effects on embryo and blastocyst development [31].

As regards mancozeb’s molecular effects in a reproductive context, increasing concentrations of this fungicide (0.001–1 µg/mL) induce a significant reduction in p53 expression levels, as suggested by Paro et al. in mouse granulosa cells [25]. This data are also confirmed by Iorio et al., whose study confirms the bidirectional linkage between p53 downregulation and higher ROS production, due to fungicide exposure [34]. Paro et al. also registered important alterations on granulosa cells morphology with reorganization of the actin cytoskeleton and acquisition of migratory competence, both in mouse then in human models [25]. Moreover, Iorio et al. highlighted a significant linkage between mancozeb exposure and mitochondrial membrane depolarization, leading to mitochondrial dysfunctions [34]. They also found that mancozeb-induced oxidative stress affects glutathione homeostasis, promoting a parallel ATP depletion [34]. In this context, ROS bioaccumulation, changes in GSH and ATP levels, p53 reduced expression, and mitochondrial potential perturbation increase the likelihood for DNA damage and apoptotic mechanisms in reproductive cells, as confirmed by different studies [49].

**Figure 2:**
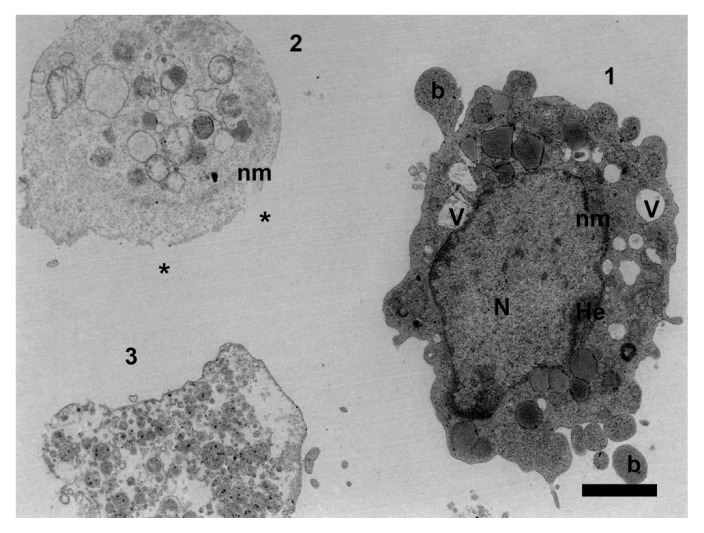
Representative transmission electron microscopy micrograph of mancozeb subcellular damage on ovarian mouse granulosa cells. Different stages of apoptotic cell death induced in vitro (0.01 mg/mL) are detectable: (1) Early stage: cell shrinkage, formation of blebs (b), dilatation and indentation of nuclear membrane (nm), heterochromatin (He) marginalization, vacuolization (V); (2) Intermediate stage: nuclear collapse, with the formation of apoptotic bodies and loss of plasma membrane integrity (*); (3) Late stage: apoptotic bodies and cell debris associated to secondary necrosis. TEM scale bar: 2 mm.

An ultrastructural overview about mancozeb’s reproductive effects on mouse granulosa cells was provided by Palmerini et al., who found nuclear membrane irregularities, intercellular contact alterations, cytoplasmic vacuolization, and chromatin condensation in mouse granulosa cells exposed to the highest levels of mancozeb [22] (Figure 2). This study confirms mancozeb gonadal toxicity, highlighting the fungicide’s power to disrupt female reproductive competence from an ultrastructural point of view.

In addition, Atmaca et al. found that mancozeb induces a progressive decline in steroid synthesis in bovine luteal cells [35]. In this study, mancozeb showed unfavorable effects on luteal cells, considered functional structures in the ovary, for their role in steroidogenesis.

As regards in vivo experimental studies, higher and moderate levels of confidence suggest that low and high doses of mancozeb or ETU decrease ovary weight [45] and enlargement [38]. On the contrary, Jacobsen et al. found no reproductive organ weight changes, especially in the uterus and ovary, in rat models exposed to mancozeb [43].

Qualitative and quantitative follicles’ alterations are also associated to mancozeb exposure: Mahadevaswami et al. found a progressive decline in health follicle counts, with a defective ovarian follicle rise, in rat models [38]. These data are also confirmed by Baligar et al. who highlight the same decline in a similar experimental model [39,40]. Liu et al. reported a significant increase in atretic follicles, in parallel with a decrease in the number of healthy follicles, in mouse models [45]. Moreover, Mahdi et al. found a remarkable number of apoptotic follicles in mancozeb-treated mouse pups, probably due to the increased ROS production in the cellular environment [47].

As regards oocyte quality, Rossi et al. found that mancozeb exposure in mouse models impairs oocyte competence by inducing a progressive egg decline, especially during ovulation [41]. Esmaiel et al., instead, suggested that mancozeb exposure has a detrimental effect on oocyte maturation and fertilization [48]; in this context, lower-quality oocytes are associated with lower fertilization rates and depleted embryonic implantation. In addition, for Liu et al., oocyte quality is damaged by mancozeb exposure; in fact, ultrastructural alterations in GV oocytes were reported, such as changes in actin expression levels, with decreased development potential of these oocytes [45].

Finally, two studies shed light on mancozeb’s potential to influence female reproductive competence, from a genetic to an epigenetic point of view. To this aim, Cuomo et al. suggested that ethylene thiourea exposure in mouse models leads to a remarkable dysregulation of ovarian aging biomarkers, affecting ovarian health [46]. Environmental exposure to ETU, in fact, induces alterations in the estrous cycle, considered the beginning of an early reproductive senescence [37,39,40,46,50]. Furthermore, Liu et al. suggested that specific histone modifications may influence the full developmental competence of mouse oocytes when exposed to mancozeb. These epigenetic modifications (e.g., H3K4me and H3K27me2), in fact, impair oocyte maturation process, compromise embryo development, and promote apoptotic pathways in the female reproductive environment [45].

Beyond experimental settings, mancozeb exerts a significant role on human public health. For 70 years, mancozeb has been applied in different agricultural contexts [21], specifically on major crops (tomato, potato, grapevine). It is likely that the increasing global demand for fruits and vegetables (also due to new eating habits, such as vegetarian and vegan diet) will boost mancozeb production in the next years, with different human side effects. A sensitive segment of the population, exposed to mancozeb, is represented by pregnant women, who use agricultural products and are at risk from their side effects. In this context, mancozeb crosses the blood–placenta [48,51,52] and the blood–milk barriers [53], compromising the development of offspring, from intrauterine to postnatal life [54].

The U.S. Environmental Protection Agency (EPA) evaluated the dietary risk of mancozeb from residues in foods, establishing the value of the population adjusted dose (PAD). The latter may be considered the reference dose for acute and chronic exposure (aPAD and cPAD). The chronic dietary risk from food was assessed by using the average consumption data for vegetables and average residue values on those foods. The number of residues varies on different fruits and vegetables, depending on the specific features of the foods. The EPA reported a cPAD value of 0.16 mg/kg/day. This is the dose at which the general population could be exposed over the course of a lifetime with no expected adverse health effects [24].

However, it must be considered that the EPA recognized a weakness of data regarding the link between mancozeb intake and reproductive impairment. To this intent, the Agency established a reduction of the cPAD by a factor of 10 (the so-called safety factor) to ensure adequate protection to women of reproductive age and for children less than six years old, setting the cPAD to 0.016 mg/kg/day [24,55].

## 5. Conclusions

As proved by this systematic review, mancozeb can be considered as a powerful threat for female reproductive competence and in vitro/in vivo models are useful to evaluate reproductive hazards. Due to its persistence and versatile profile, this fungicide compromises female reproduction in different ways. In this context, mancozeb is an epigenetic hazard and a powerful environmental pollutant, which interacts with female reproductive phenotypes, changing directly or indirectly the inner molecular and cellular balances.

For this reason, even if the available evidence gives more insight on mancozeb gonadal toxicity, further studies are required for a complete etiologic and epidemiologic understanding of this health concern. However, this review may contribute to fulfil the gap in risk assessment of mancozeb reproductive impairment and may also be useful for government agencies in normative decision-making on environmental and occupational health.

## Figures and Tables

**Table 1:** Confidence ratings for mancozeb health effects.

Level of Confidence for Health Effects		
**++++**	High Confidence	Association between substance exposure and the outcome. The true effect is highly likely to be reflected in the apparent relationship.
**+++**	Moderate Confidence	Association between substance exposure and the outcome. The true effect may be reflected in the apparent relationship.
**++**	Low Confidence	Association between substance exposure and the outcome. The true effect may be different from the apparent relationship.
**+**	Very Low Confidence	Association between substance exposure and the outcome. The true effect is highly likely to be different from the apparent relationship.

**Table 2:** In vitro studies: mancozeb exposure and reproductive outcomes. Confidence ratings in the body of evidence ratings: High Confidence (++++) in the association between mancozeb exposure and female reproductive outcomes. The true effect is highly likely to be reflected in the apparent relationship. Moderate Confidence (+++) in the association between mancozeb exposure and female reproductive outcomes. The true effect may be reflected in the apparent relationship. Low Confidence (++) in the association between mancozeb exposure and female reproductive outcomes. The true effect may be different from the apparent relationship. Very Low Confidence (+) in the association between mancozeb exposure and female reproductive outcomes. The true effect is highly likely to be different from the apparent relationship.

Author (Year)	Type of Cell/Tissue	Compound (Daily Dose)	Incubation	Outcomes	Confidence
**Fejes et al. (2002)**	288 chicken embryos	80% mancozeb containing formulation (dithane M-45)	19 days	No toxic effect on embryos. Increased mortality in embryos in combination mixtures.	++
**Greenlee et al.** **(2004)**	Mice embryos	Low-doses of agrochemicals mancozeb (0.003 µg/mL)	96 h	Reduced development of mouse blastocysts. Increased apoptosis during blastocyst formation	+++
**Abdoon et al.** **(2011)**	Buffalo oocytes	Mancozeb (1.0, 2.5, and 5.0 µg/mL)	24 h	In vitro maturation: mancozeb affects cytoplasmic and nuclear maturation. Dose-dependent oocyte degeneration. Fragmented cytoplasm and broken zona pellucida. In vitro fertilization: lower embryo development to morula and blastocyst stage. Fragmented and degenerated cytoplasm in all the exposed embryos.	+++
**Paro et al.** **(2012)**	Mouse granulosa cells	Increasing concentrations of mancozeb (0.001–1 µg/mL)	1, 24, and 36 h	Mouse: morphology changes; migration pattern; p53 reduced expression; no changes in apoptosis. Human: morphology changes; p53 reduced expression.	++++
**Iorio et al.** **(2015)**	Mouse granulosa cells	Mancozeb (0.01 µg/mL)	36 h	p53 reduced expression. Depolarized mitochondrial membrane potential. Decreased ATP levels. Decreased glutathione levels (GSH). Increased reactive oxygen species (ROS).	++++
**Palmerini et al.** **(2018)**	Mouse granulosa cells	Increasing concentrations of mancozeb (0.001, 0.01, 0.1, and 1 µg/mL)	36 h	Dose-dependent toxicity of mancozeb on mouse granulosa cells.	++++
**Atmaca et al.** **(2018)**	Bovine luteal cells	Mancozeb (0.01, 0.1, and 1 µM)	4 days	Mancozeb exposure (1 µM) induces a significant decline (day 3 and 5) in steroidogenesis, compared to controls.	+++

**Table 3:** In vivo studies: mancozeb exposure and reproductive outcomes. Confidence ratings in the body of evidence ratings: High Confidence (++++) in the association between mancozeb exposure and female reproductive outcomes. The true effect is highly likely to be reflected in the apparent relationship. Moderate Confidence (+++) in the association between mancozeb exposure and female reproductive outcomes. The true effect may be reflected in the apparent relationship. Low Confidence (++) in the association between mancozeb exposure and female reproductive outcomes. The true effect may be different from the apparent relationship. Very Low Confidence (+) in the association between mancozeb exposure and female reproductive outcomes. The true effect is highly likely to be different from the apparent relationship.

Author (Year)	Experimental Animal	*n*	Compound (Route)	Daily Dose	Duration	Outcomes	Ratings
**Khera et al. (1973)**	Adult nulliparous female Wistar rats, New Zealand White rabbits	209 rats, 33 rabbits	Ethylene thiourea (ETU; oral)	0, 5, 10, 20, 40, or 80 mg/kg/day	Rats Group 1: 21–42 (before gestation), 1–15 days (gestation) Group 2: 6–15 (gestation) Group 3: 7–20 days (gestation) Rabbits: 30 days (gestation)	No changes in the number of viable fetuses or in fetal death. 80 mg/kg of ETU have no significant effect before and during pregnancy.	++++
**Castro et al. (1999)**	Wister rats	120	Mancozeb (mixed in diet)	0, 2000–3000 ppm	Group A: 1–6 (gestation days) Group B: 6–15 (gestation days)	No changes in estrous cycle during pregnancy. No changes in the number of live pup births.	+++
**Mahadevaswami et al. (2000)**	Female Wistar virgin rats	36	Mancozeb (oral)	500, 600, 700, and 800 mg/kg/day	15 (before gestation days)	Decrease in ovary enlargement (700 and 800 mg/kg/day). No changes in estrous cycle (500 mg/kg/day). Decline in health follicle counts and a defective ovarian follicle rise (800 mg/kg/day).	++++
**Baligar et Kaliwal** **(2001)**	Wister virgin rats	40	Mancozeb (oral)	500, 600, 700, and 800 mg/kg/day	30 days	Decline in number of estrous cycle and healthy follicles, with changes in proestrus, estrus. and metestrus phases.	+++
**Baligar et Kaliwal** **(2004)**	Female virgin albino rats	70	Mancozeb (75% wettable powder, olive oil; oral)	700 mg/kg/day	5, 10, 20, or 30 days (before gestation)	Alteration in diestrus and estrous cycle. Decline of healthy follicle numbers. Atretic follicles increase.	+++
**Rossi et al.** **(2006)**	Swiss CD-1 female mice	25	Mancozeb (sesame oil; oral)	50 and 500 mg/kg	Gestation day 2, pup day 20	Eggs decline (ovulation) and fertilizability decreases.	+++
**Hass et al.** **(2012)**	Nulliparous time-mated young adult female Wistar rats	198	Mancozeb (mixture composition; gavage)	6.25 and 25 mg/kg/day	7–21 (gestation day) 1–16 (pup day)	Longer gestation period (highest concentration).	++++
**Jacobsen et al.** **(2012)**	Nulliparous time-mated young adult female Wistar rats	198	Mancozeb (mixture composition; gavage)	6.25 and 25 mg/kg/day	Gestation day 7, pup day 16	No reproductive organ weight alterations (uterus and ovary).	++++
**Jacobsen et al.** **(2010)**	Nulliparous time-mated young adult female Wistar rats	80	Mancozeb (mixture composition; gavage)	6.25, 12.5, 18.75, 25.0, and 31.25 mg/kg/day	Gestation day 7–day before expected birth (GD21)	Higher perinatal pup mortality and impaired parturition.	++++
**Liu et al.** **(2017)**	CD-1 mice (4–6 weeks old)	240	Mancozeb (oral)	800 mg/kg/day	4 weeks	Mancozeb reduces litter size and weight. Decreased ovary weight. Increased atretic follicles and decreased normal oocytes. Ultrastructural alterations in GV oocytes. Decrease in the number of pronuclei and two-cells of parthenogenetic activated oocytes. Changes in actin expression levels. Increase in apoptosis and in ROS production. Abnormal mitochondrial distribution and mitochondrial membrane alterations. Epigenetic modifications.	++++
**Cuomo et al.** **(2018)**	CD-1 mice	20	Ethylene thiourea (ETU; drinking water)	0.1, 1, and 10 mg/kg/day	From conception (through mothers) to postnatal 21 days.	ETU influences ovarian aging biomarkers at all doses.	++++
**Mahdi et al.** **(2019)**	First-generation (F1) mouse female pups	36	Mancozeb (oral gavage)	500 mg/kg	From day 2 of pregnancy to postnatal 20 days.	Apoptotic follicles. Remarkable germ cells depletion in gonads.	++++
**Esmaiel et al.** **(2019)**	First-generation (F1) female pups	60	Mancozeb (oral gavage)	500 mg/kg (olive oil; mothers)	From day 2 of pregnancy to postnatal 21 days.	Reduction of number of collected oocyte. Comprised oocyte maturation, fertilization, implantation, and fecundity rate. Comprised embryo development.	++++
